# Diagnostic Accuracy of Novel AI-Based Software in the Detection of Dental Caries on Bitewing and Intraoral Periapical Radiographs

**DOI:** 10.3390/diagnostics16162566

**Published:** 2026-08-14

**Authors:** Bhavana Sujanamulk, Ahmed A. Almeshari, Bharani Krishna Takkella, Mohammed A. Barayan, Enas Ahmed Elamin, Khadijah Mohideen, Balwinder Singh

**Affiliations:** 1 Oral & Maxillofacial Surgery and Diagnostic Sciences, Faculty of Dentistry, Najran University, Najran P.O. Box 1988, Saudi Arabia; bsvjanamulk@nu.edu.sa (B.S.); kmohideen@nu.edu.sa (K.M.); 2 Oral Medicine & Radiology, Drs. Sudha and Nageswararao Siddhartha Institute of Dental Sciences, Vijayawada 520001, India; bharanikrishna3@gmail.com; 3 Oral & Diagnostic Sciences, Faculty of Dentistry, King Abdulaziz University, Jeddah 21589, Saudi Arabia; mbarayan@kau.edu.sa; 4 Department of Preventive Dentistry, Faculty of Dentistry, Najran University, Najran P.O. Box 1988, Saudi Arabia; enas.elamin8@gmail.com; 5 Oral Medicine & Radiology, Sri Guru Ram Das Institute of Dental Sciences & Research, Amritsar 143001, India; dentalmanuscript@gmail.com

**Keywords:** artificial intelligence, dental caries, deep learning, diagnostic accuracy, dental radiology, computer-aided diagnosis

## Abstract

**Background:** Dental caries detection using intraoral radiographs is essential for early diagnosis but may be affected by observer variability. Artificial intelligence (AI)-based systems have emerged as potential tools to improve diagnostic consistency. This study evaluated the diagnostic accuracy of a deep learning-based AI system **(Better Diagnostics Caries Assist (BDCA) Version 1.0)** for detecting dental caries on bitewing (BW) and intraoral periapical (IOPA) radiographs, and examined its performance across demographic, technical, and lesion-based subgroups. **Methods:** A retrospective validation study was conducted using anonymized digital BW and IOPA radiographs with expert-defined ground truth at the tooth-surface level. The AI software independently analyzed each image to identify carious lesions. Sensitivity, specificity, positive predictive value (PPV), and negative predictive value (NPV) were calculated with 95% confidence intervals. Generalized estimating equations were applied to adjust for the clustering of multiple surfaces per image. Subgroup analyses were performed by age, sex, digital sensor type, and lesion category. **Results:** The AI system demonstrated high diagnostic accuracy for both modalities. For BW radiographs, sensitivity was 0.892 and specificity was 0.995, while for IOPA radiographs sensitivity was 0.882 and specificity was 0.991. NPVs exceeded 0.99 for both modalities. Across age groups, BW sensitivity ranged from 0.881 to 0.901 and IOPA sensitivity from 0.854 to 0.906, with consistently high specificity (>0.989). Sex-based differences were minimal. Sensor-wise analysis showed sensitivity ranging from 0.833 to 0.933 for BW and 0.828 to 0.933 for IOPA, while specificity remained above 0.984 for all sensors. Detection performance was comparable for primary (sensitivity 0.883) and secondary caries (0.879), although PPV was slightly lower for secondary lesions. The lower AUC indicated reduced accuracy in lesion identification in the absence of BDCA v 1.0, the difference between BDCA v1.0 and Ground truth was 0.042 at 95% CI 0.030 to 0.055, and the difference was also statistically significant with (*p* ≤ 0.001). **Conclusions:** The evaluated AI system demonstrated excellent and consistent performance for detecting dental caries on both BW and IOPA radiographs across demographic groups, sensor technologies, and lesion types, supporting its potential role as a reliable decision support tool in dental radiographic interpretation.

## 1. Introduction

Dental caries remains one of the most prevalent chronic diseases worldwide, affecting individuals across all age groups and representing a major public health burden. According to the World health organization (WHO) the Global Burden of Disease Study, untreated caries in permanent teeth is among the most common health conditions globally, highlighting the need for accurate and early diagnosis to prevent progression and tooth loss [1]. The early detection of caries is critical because timely intervention can halt disease progression, preserve tooth structure, and reduce the need for invasive restorative procedures [2].

Radiographic imaging plays a fundamental role in the detection of dental caries, particularly for lesions that are not clinically visible. Bitewing (BW) radiographs are considered the standard method for detecting proximal caries due to their ability to visualize interproximal enamel and dentin with minimal anatomical overlap. In contrast, intraoral periapical (IOPA) radiographs are commonly used for assessing periapical pathology and root structures, but are also frequently used in routine dental practice to evaluate caries when bitewing imaging is unavailable [3,4]. Despite their widespread use, radiographic interpretation is inherently subjective and depends heavily on clinician experience, image quality, and diagnostic conditions. Studies have shown substantial inter- and intra-observer variability in radiographic caries detection, which can lead to both missed lesions and false-positive diagnoses [5].

Advances in artificial intelligence (AI), particularly deep learning techniques, have created new opportunities for improving diagnostic accuracy in medical and dental imaging. Convolutional neural networks (CNNs), a class of deep learning models designed for image analysis, have demonstrated remarkable performance in recognizing complex visual patterns and detecting pathological changes in radiographs [6]. Over the past decade, AI-based diagnostic systems have shown performance comparable to or exceeding that of human experts in several medical imaging domains, including dermatology, ophthalmology, and radiology [7,8]. These successes have stimulated growing interest in applying AI technologies to dental radiographic interpretation.

In dentistry, AI applications have expanded rapidly in areas such as caries detection, periodontal bone loss assessment, tooth segmentation, and treatment planning. Several studies have reported promising results for AI-based caries detection systems. Schwendicke et al. demonstrated that deep learning models could achieve diagnostic accuracy comparable to experienced dentists in detecting proximal caries on bitewing radiographs [9]. Similarly, Albano et al. reported that a CNN-based system achieved high sensitivity and specificity for detecting dental caries on radiographic images, supporting the feasibility of automated caries screening [10]. A systematic review and meta-analysis by Hung et al. further confirmed that machine learning models can achieve pooled sensitivity approaching 0.89 and specificity exceeding 0.90 for radiographic caries detection, suggesting that AI tools may significantly enhance diagnostic reliability [11].

Despite these advances, several gaps remain in the current literature. First, many previous studies have focused primarily on a single imaging modality, most commonly bitewing radiographs, with limited evaluation of AI performance across multiple radiographic types used in routine dental practice. Since clinicians frequently rely on both BW and IOPA radiographs, evaluating AI performance across both modalities is essential for assessing real-world clinical applicability. Second, the robustness of AI diagnostic systems across different patient demographics, imaging sensors, and lesion types has not been extensively explored. Variations in age, restorative status, or imaging hardware may influence radiographic appearance and potentially affect algorithm performance [12]. Therefore, comprehensive validation across diverse subgroups is necessary before AI tools can be reliably implemented in routine clinical workflows.

Another important consideration is that most AI studies emphasize model performance in controlled experimental datasets, whereas practical clinical adoption requires the demonstration of consistent performance across heterogeneous imaging conditions. Validation studies incorporating multiple sensor types, real-world radiographs, and subgroup analyses are therefore crucial for establishing the generalizability and clinical reliability of AI systems [13]. Furthermore, evaluating AI performance at both the surface level and image level can provide a more nuanced understanding of diagnostic capability and clinical utility.

Given these considerations, the present study was undertaken to evaluate the diagnostic accuracy of a deep learning-based AI system in detecting dental caries on both bitewing and intraoral periapical radiographs. The study aimed to assess overall performance metrics, including sensitivity, specificity, positive predictive value, and negative predictive value, and to analyze performance across demographic subgroups, sensor types, and lesion categories. By providing comprehensive validation across multiple imaging modalities and technical conditions, this investigation seeks to contribute to the growing evidence supporting the role of AI-assisted radiographic interpretation in enhancing diagnostic precision and improving patient care in dentistry.

## 2. Methodology

### 2.1. Study Design and Reporting Standards

**Figure 1:**
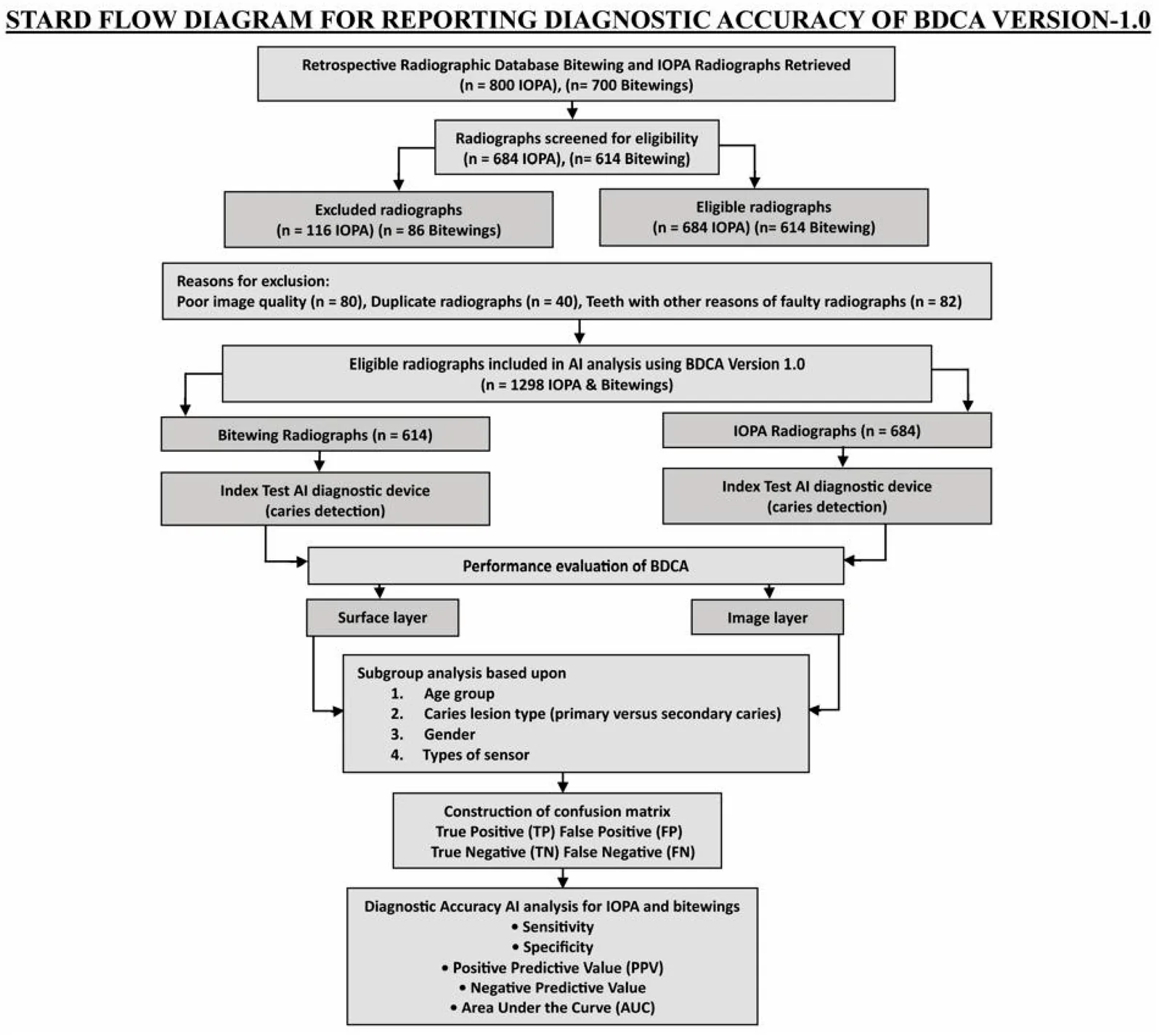
STARD flow diagram for reporting diagnostic accuracy of BDCA.

This investigation was conducted as a retrospective diagnostic accuracy validation study to evaluate the standalone performance of an artificial intelligence (AI)-based software system, **Better Diagnostics Caries Assist (BDCA) version 1.0**, for detecting dental caries on intraoral radiographs. The study assessed the ability of the AI system to automatically identify and localize carious lesions on bitewing (BW) and intraoral periapical (IOPA) radiographs without clinician assistance. The design followed established methodological principles for diagnostic test evaluation and complied with the Standards for Reporting Diagnostic Accuracy Studies (STARD 2015) recommendations, as well as emerging guidance for AI-based clinical prediction and imaging studies to ensure transparency, reproducibility, and methodological rigor (Figure 1) [14,15].

### 2.2. Study Setting and Data Source

The study used retrospectively collected anonymized digital intraoral radiographs obtained from routine clinical practice. Images represented real-world diagnostic conditions and included a wide demographic range across age groups and gender. Multiple commercially available digital sensor systems were represented in the dataset, including Apex Digital Sensor, Carestream RVG 5200, Dentimax Pro Sensor, Dexix Titanium, Vatech Ez Sensor HD, Jazz Digital Imaging Sensor and SHICK 33, thereby allowing for the evaluation of performance across different acquisition technologies. The inclusion of heterogeneous imaging sources is recommended in AI validation studies to improve external validity and minimize spectrum bias [12].

## 3. Eligibility Criteria and Image Selection

### 3.1. Inclusion Criteria

1. All the digital bitewing and intraoral periapical radiographs obtained from the institutional radiographic databases;
2. Radiographs of permanent teeth with diagnostic image quality;
3. Radiographs showing surface caries or early caries either proximal or occlusal surface for caries assessment;
4. Radiographs which were compatible with our BDCA software (version 1.0) were all included.

### 3.2. Exclusion Criteria

1. Radiographs with poor diagnostic quality;
2. Radiographs containing significant artifacts considered to be faulty radiographs;
3. Images with incomplete crown where caries is not properly visible;
4. Duplicate radiographs or repeat images of the same tooth taken during the study period were all excluded.

Only diagnostic-quality BW and IOPA radiographs showing permanent dentition and allowing for the adequate visualization of relevant tooth surfaces were included. Radiographs with severe motion blur, improper exposure, incomplete anatomical visualization, or duplicated records were excluded.

Images previously used in algorithm training or internal development were not included in the validation dataset to prevent information leakage and ensure unbiased performance estimation. The final dataset included BW and IOPA radiographs, comprising multiple tooth surfaces evaluated at the surface level. The details are enclosed in Table 1 for both IOPA and bitewing radiographs.

### 3.3. Ground Truth Annotation and Reference Standard

The radiologists who actually established the reference standard were involved in the evaluation of radiographs. No additional radiologists were involved in the assessment of carious lesions. The assessment was performed independently, and the BDCA software 1.0 was evaluated against this reference standard, which was set by **four experienced radiologists**, each with **10 yrs of experience in oral and maxillofacial radiology/radiographic interpretation**. The radiologists independently evaluated the radiographic images for the presence or absence of carious lesions. When there was disagreement between them, a consensus was established through another experienced radiologist with more than 15 yrs, and the conclusion was reached. Expert consensus evaluation of radiographic findings is widely accepted as a practical reference standard in diagnostic imaging studies where histological confirmation is not feasible [16].

**Figure 2:**
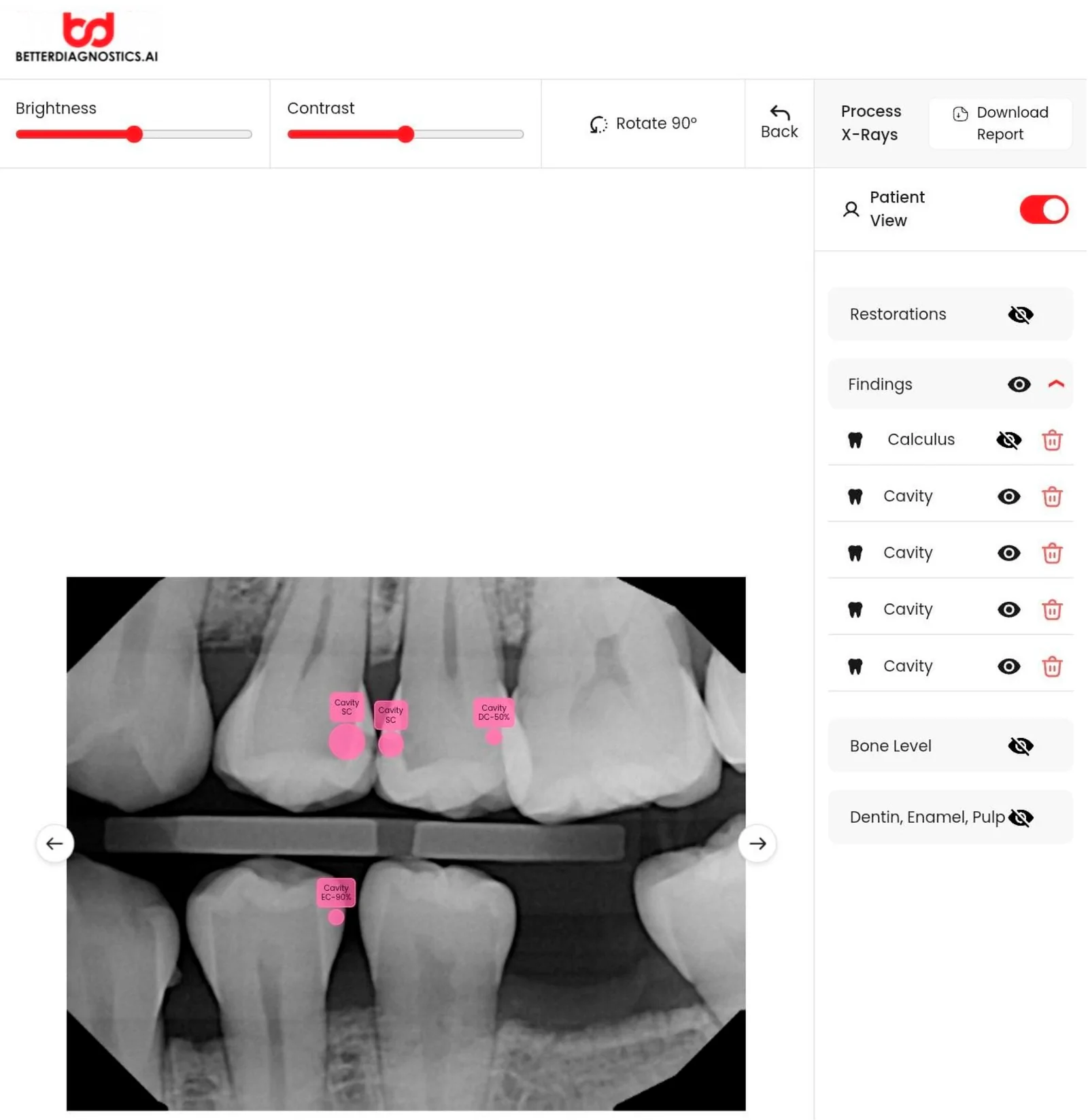
BDCA software showing the carious lesion on Bitewing Radiograph.

**Figure 3:**
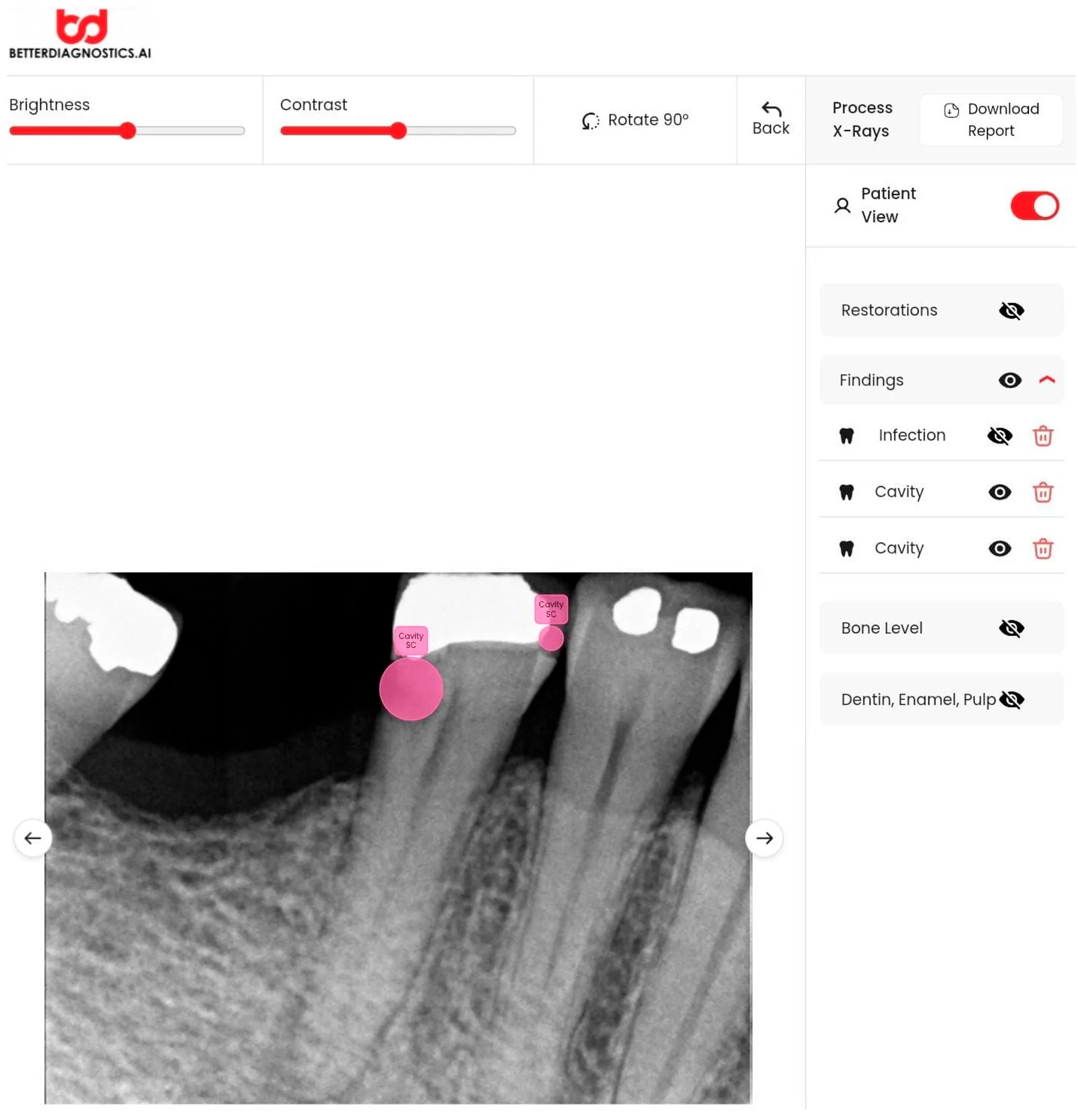
BDCA software showing the carious lesion on Intraoral radiograph radiograph.

Annotation procedures followed standardized diagnostic criteria for radiographic caries detection, ensuring consistency across evaluators. The finalized annotated dataset served as the ground truth for comparison with AI-generated predictions (Figure 2 and Figure 3).

### 3.4. Artificial Intelligence System and Image Processing

**BDCA v1.0** is a deep learning-based AI system designed for the automated analysis of dental radiographs. The software utilizes convolutional neural networks (CNNs), which are widely used in medical imaging because of their ability to learn the spatial hierarchies of image features and detect subtle structural variations associated with pathology [6,17].

It is integrated with the features **Model architecture (network depth, layer specifications):** Model Architecture (BDCA): YOLOv12 (Backbone + PAN-FPN Neck + Decoupled Detection Head). The training parameters of this software includes core training parameters (Learning Rate Schedule): Initial Learning Rate = 0.01, Cosine Annealing Learning Rate Scheduler with 5 Warm-up Epochs, Batch Size: 32, Loss Function: Complete IoU (CIoU) Loss for Bounding Box Regression, Binary Cross-Entropy (BCE) Loss for Classification, and Binary Cross-Entropy (BCE) Loss for Objectness Prediction, Epoch Duration: 3000 Epochs, along with **Threshold optimization procedures**, Optimization Algorithm: Stochastic Gradient Descent (SGD) with Momentum = 0.937 and Weight Decay = 0.0005. **The data augmentation** for BDCA component included Rotation: ±7° to ±10° Translation: Up to 10%, Scale (Zoom): 0.9–1.1×, Brightness Adjustment: ±15–20%, Contrast Adjustment: ±15–20%, Gamma Correction: 0.8–1.2, CLAHE: Clip Limit 2–3, Tile Grid 8 × 8, Gaussian Noise: σ = 0.005–0.02, Gaussian Blur: 3 × 3 kernel, σ ≤ 1.0 (*p* = 0.2), Random Crop + Resize: Crop 90–100%, then resize to original size.

Radiographs were input into the AI software in their native digital format. Prior to analysis, standard preprocessing procedures such as the normalization of image intensity, resizing, and format harmonization were applied automatically by the software pipeline. The AI system processed each image independently and generated outputs indicating suspected lesion locations and classification probabilities. These outputs were then converted into binary diagnostic predictions according to predefined decision thresholds specified in the validation protocol.

### 3.5. Outcome Measures

The primary outcome measures were sensitivity, specificity, positive predictive value (PPV), and negative predictive value (NPV). Sensitivity represented the proportion of true carious surfaces correctly identified by the AI system, whereas specificity represented the proportion of non-carious surfaces correctly classified. PPV and NPV were calculated to estimate the reliability of positive and negative predictions in a clinical context.

Performance evaluation was conducted at both the surface level and the image level. Surface-level analysis assessed lesion detection accuracy for individual tooth surfaces, while image-level analysis evaluated whether at least one lesion was correctly detected within a radiograph.

Subgroup analyses were conducted to examine diagnostic performance across age groups, sex, sensor types, and lesion types (primary versus secondary caries).

### 3.6. Statistical Analysis

All statistical analyses were conducted using (Python 3.10.0. python version) within a reproducible computational environment. Data processing and descriptive statistics were performed using pandas and NumPy, while diagnostic performance metrics were computed using scikit-learn and SciPy. Generalized estimating equation (GEE) models implemented in stats models were used to account for the clustering of multiple tooth surfaces within individual radiographs and to obtain adjusted 95% confidence intervals.

Diagnostic accuracy was evaluated by calculating sensitivity, specificity, positive predictive value, negative predictive value, and overall accuracy, based on the comparison of AI predictions with the expert reference standard. Confusion matrices were generated to summarize classification outcomes. Where applicable, the area under the curve (AUC) values were computed to assess discriminative performance. Subgroup analyses were performed to evaluate diagnostic performance across predefined categories, including age group, sex, imaging sensor type, and lesion category (primary versus secondary caries). Confidence intervals for proportions were calculated using binomial methods, and clustered estimates were derived from GEE models to ensure valid inference in the presence of correlated observations. The analysis workflow was version-controlled to ensure reproducibility, and all scripts were executed using standardized library versions within the Python environment.

### 3.7. Bias Control and Validation Integrity

To minimize bias, the dataset used for validation was independent of the training dataset, and AI predictions were generated without access to ground truth annotations. Radiologist reviewers establishing the reference standard were blinded to AI outputs during annotation.

### 3.8. Ethical Considerations

All radiographic images were anonymized prior to analysis to protect patient confidentiality. The study used retrospective imaging data and involved no direct patient intervention. The informed consent was waived as the study was retrospective in nature, and only fully anonymized data were used for the study. Here, the required data to assess the accuracy of AI software were provided by the Faculty of dentistry, Najran University, and Drs Sudha Nageswararao Siddhartha institute of dental sciences [18].

## 4. Results

Table 1 represents the dataset variables to assess the BDCA AI software for IOPA and bitewing which includes total no of participants, total no of radiographs, total no of tooth surfaces involved and absolute no of True Positives (Carious tooth) True Negative (Non Carious tooth) among both intraoral radiographs.

Table 2 shows that both bitewing (BW) and intraoral periapical (IOPA) images demonstrated high diagnostic accuracy for caries detection. BW images showed slightly higher sensitivity (0.892) and specificity (0.995) compared with IOPA images (0.882 and 0.991, respectively). Negative predictive value (NPV) was extremely high for both modalities (>0.99), indicating excellent reliability in ruling out disease when lesions were absent. Positive predictive values (PPV) were also comparable, suggesting both imaging techniques provide robust confirmation when caries is detected. Overall, BW images showed marginally better diagnostic performance.

As shown in Table 3, diagnostic performance remained consistently high across all age groups for both modalities. For BW images, sensitivity ranged from 0.881 to 0.901, with slightly higher sensitivity observed in patients aged >60 years. Specificity remained uniformly high (~0.994–0.995) across age groups.

For IOPA images, the 18–40-year group showed the highest sensitivity (0.906), while sensitivity declined slightly in older age groups (>60 years: 0.854). Despite this variation, specificity and predictive values remained consistently high in all age strata. These findings indicate that age did not substantially influence diagnostic reliability, although younger patients showed slightly better detection with IOPA imaging.

Table 4 demonstrates minimal sex-related variation in diagnostic performance. For BW images, females showed slightly higher sensitivity (0.910) compared with males (0.875), while specificity was nearly identical in both sexes (~0.994–0.995). Similarly, for IOPA images, males had marginally higher sensitivity (0.901) than females (0.866), but specificity and predictive values were comparable. Overall, these findings suggest that sex had negligible impact on diagnostic accuracy, and both modalities performed consistently in male and female patients.

Table 5 reveals moderate variation in diagnostic performance across different digital sensors. For BW images, Apex, Dexix, and Vatech sensors showed high sensitivity (>0.92), whereas Dentimax showed comparatively lower sensitivity (0.833) but the highest specificity (0.998). For IOPA images, Jazz sensor demonstrated the highest sensitivity (0.933), followed closely by Apex (0.925). Dexix showed the lowest sensitivity among IOPA sensors (0.828). Specificity remained high (>0.98) for all sensors in both modalities, indicating excellent ability to correctly identify non-carious surfaces regardless of sensor type.

As presented in Table 6, IOPA images showed similar sensitivity for primary (0.883) and secondary decay (0.879), indicating consistent detection capability across lesion types. However, PPV was higher for primary decay (0.837) compared with secondary decay (0.758), suggesting greater certainty in identifying primary lesions.

**Figure 4:**
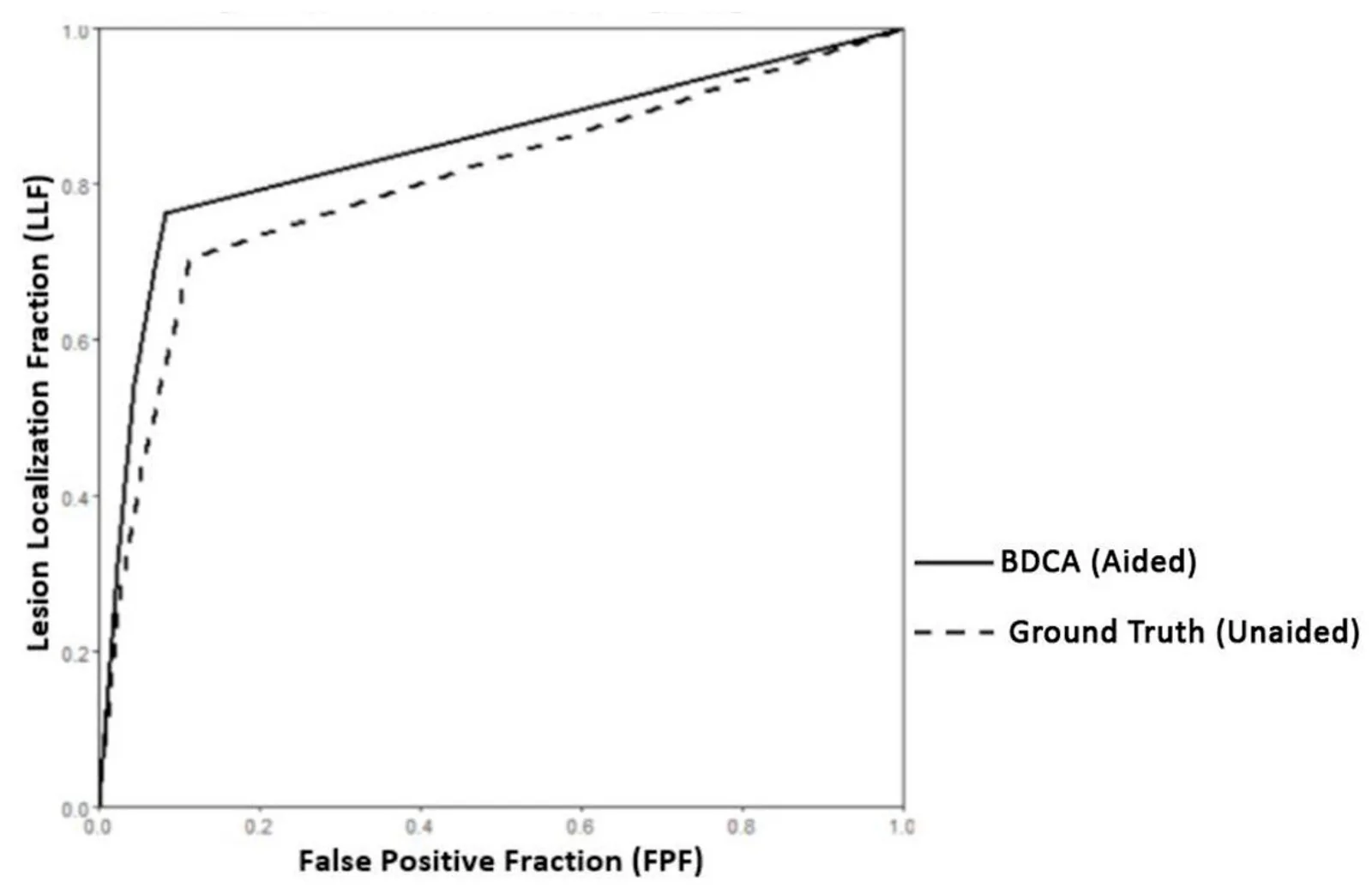
AUC curve for Bitewing image.

This primary endpoint for BW images to evaluate the BDCA (AI assisted) versus Ground Truth (without AI software) through AUC analysis is described in (Figure 4). In the BDCA group: The AUC for the software group was 0.848, with a 95% CI ranging from 0.824 to 0.867. This suggests that when BDCA v1.0 was used along with the interpretation of radiologist, the readers’ ability to accurately identify targets in the images was higher. Likewise, for the oral radiologists who were considered as ground truth, the AUC value was 0.806, with the 95% CI between 0.786 and 0.820. This lower AUC indicated a reduced accuracy in target identification without BDCA v1.0. The difference between the BDCA and Ground Truth AUC was 0.042, with a 95% CI of 0.030 to 0.055. The statistical significance of this difference was confirmed by a *p* ≤ 0.001, demonstrating that the improvement of reader’s performance with BDCA v1.0 assisted was statistically significant and higher compared to without using BDCA software.

**Figure 5:**
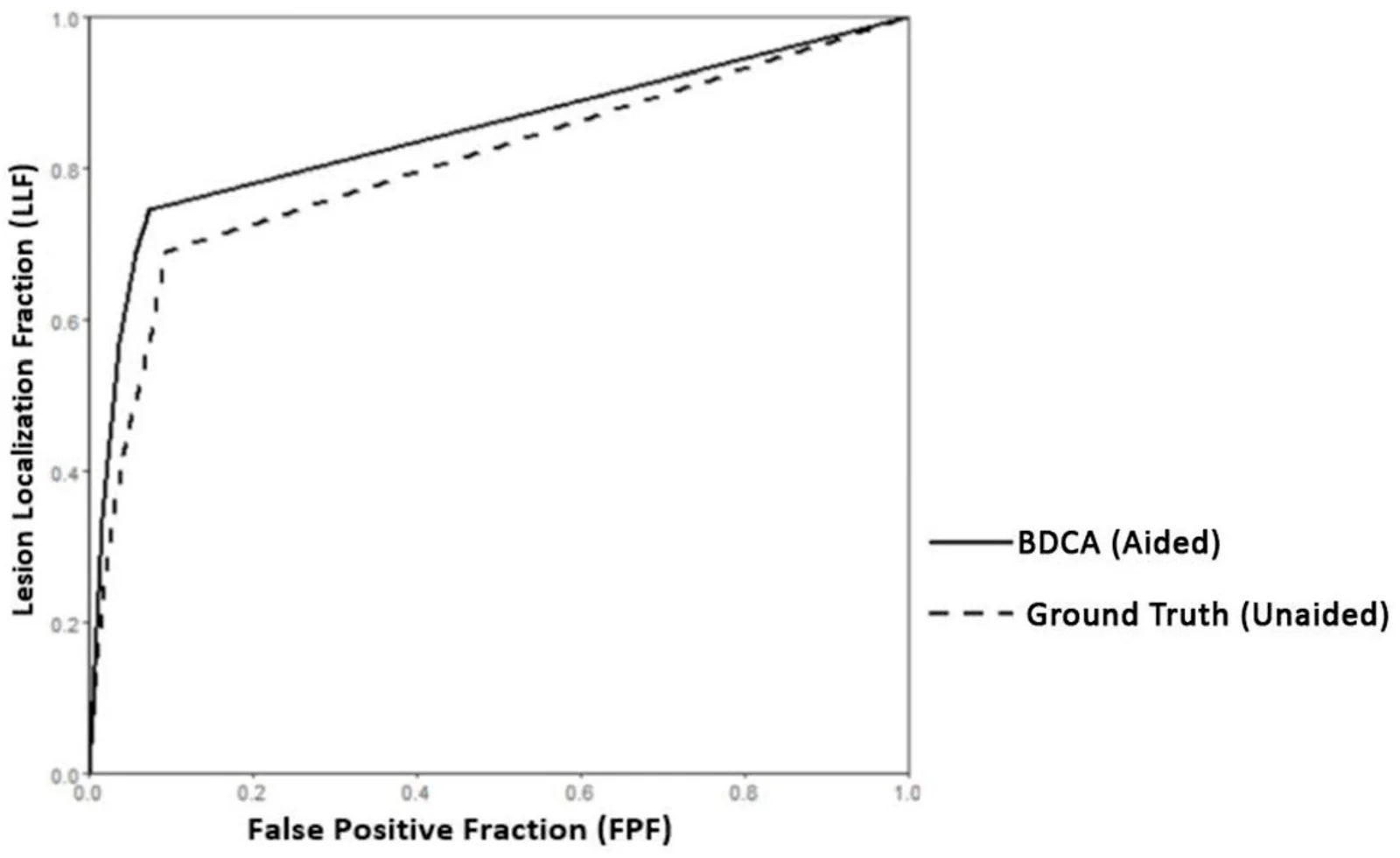
AUC curve for IOPA images.

This primary endpoint for IOPA images to evaluate BDCA (AI assisted) versus Ground Truth (without AI software) through AUC analysis is represented in Figure 5. In BDCA group i.e.,: AI assisted the AUC for the software group was 0.845, with a 95% CI from 0.819 to 0.860. This indicated accuracy in identifying targets with the assistance of BDCA v1.0. Similarly, the Oral radiologist who were considered as ground truth, the AUC value was 0.807, with a 95% CI ranging from 0.781 to 0.819. This value reflects lower accuracy in target identification when BDCA v1.0 was not used. The difference in AUC between BDCA and Ground truth was 0.039, with a 95% CI of 0.030 to 0.049. The *p* ≤ 0.001 demonstrates accuracy with BDCA v1.0 (AI assisted -aided conditions was statistically significant.

## 5. Discussion

The present study evaluated the diagnostic accuracy of an AI-based system (BDCA 1.0) Better Diagnostics Caries Assist (BDCA) Version 1.0 for detecting dental caries on BW and IOPA radiographs. The results demonstrated consistently high sensitivity, specificity, and predictive values across both imaging modalities, demographic subgroups, sensor types, and lesion categories. Overall, BW images showed marginally higher diagnostic performance than IOPA images, although both modalities achieved excellent specificity and negative predictive values. These findings support the growing body of evidence that deep learning-based AI systems can reliably assist in radiographic caries detection and may improve diagnostic consistency in clinical dental practice [19,20,21].

The findings of the present study are consistent with previous investigations evaluating AI-based caries detection on intraoral radiographs. Lee et al. developed a deep learning convolutional neural network (U-Net architecture) for detecting dental caries on bitewing radiographs and demonstrated that the model achieved accurate detection performance and could meaningfully assist clinicians in accurate radiographic diagnosis. Their study showed that deep learning systems can reliably identify radiographic density variations associated with carious lesions and improve diagnostic decision-making [22].

Similarly, a recent systematic review and meta-analysis of AI models for caries detection on bitewing radiographs reported pooled sensitivity of approximately 0.87 and specificity of 0.89, indicating that AI approaches provide clinically acceptable diagnostic accuracy across multiple studies, although methodological heterogeneity and risk of bias were noted [23].

In comparison, the present study demonstrated slightly higher specificity and comparable sensitivity across both bitewing and intraoral periapical radiographs, suggesting that the evaluated AI system performs at least as well as previously reported models. One possible explanation for this difference is the inclusion of diverse sensor types and real-world radiographic data, which may improve algorithm robustness. Prior literature emphasizes that dataset diversity, annotation quality, and training size strongly influence AI diagnostic performance in dental imaging [9].

One important observation from this study is that bitewing radiographs demonstrated slightly higher sensitivity and specificity than intraoral periapical radiographs. This difference is consistent with traditional radiographic principles. Bitewing imaging provides superior visualization of proximal surfaces and early enamel lesions compared with periapical radiographs, which are optimized primarily for periapical and root assessment [3]. Previous research has consistently demonstrated that BW radiographs outperform IOPA images in detecting proximal caries due to reduced anatomical overlap and improved contrast in the interproximal region [5]. For example, Kuhnisch et al. highlighted that bitewing radiography remains the gold standard for proximal caries detection in routine dental practice because of its higher diagnostic yield compared with periapical views [4]. The marginally better AI performance on BW images in this study likely reflects these inherent imaging advantages rather than limitations of the algorithm itself.

Despite this difference, the AI system demonstrated robust performance on IOPA radiographs as well, suggesting that the algorithm effectively generalizes across imaging modalities. This generalizability is crucial for clinical implementation because dentists often rely on both BW and IOPA radiographs depending on patient needs. Previous studies have emphasized that AI models trained on diverse imaging datasets show improved robustness and reduced modality-specific bias [12]. The high performance across modalities observed here supports the adaptability of the AI system and suggests its potential utility in varied clinical workflows.

Subgroup analyses by age demonstrated stable diagnostic performance across all age categories, with only minor variations in sensitivity. Younger patients showed slightly higher sensitivity for IOPA images, while older patients exhibited comparable or slightly improved detection on BW images. These differences may be explained by anatomical and radiographic factors. Younger patients often present with clearer enamel–dentin contrast and fewer restorations, facilitating lesion detection both for clinicians and AI systems. In older populations, restorative materials, cervical wear, and root exposure may complicate radiographic interpretation [2]. Nonetheless, the consistently high specificity and predictive values across age groups indicate that the AI system maintained reliable diagnostic performance regardless of patient age. Similar findings have been reported in recent AI validation studies, which found minimal variation in model accuracy across demographic categories when training datasets included sufficient diversity [24].

Sex-based subgroup analysis in the present study revealed negligible differences in diagnostic performance between males and females. This observation is expected, as radiographic caries detection depends primarily on anatomical and pathological features rather than sex-specific biological differences. Previous diagnostic imaging studies have also reported no clinically significant variation in radiographic caries detection accuracy between sexes [4]. The uniformity observed here further supports the algorithm’s robustness and suggests that demographic bias is unlikely to affect its performance.

The analysis of different digital sensor types revealed moderate variation in sensitivity, while specificity remained consistently high across sensors. Some sensors demonstrated slightly superior sensitivity, which may be attributable to the differences in image resolution, dynamic range, or noise characteristics. High-resolution sensors generally provide better contrast and structural detail, which can enhance both the clinician and AI detection of early lesions [25]. However, the fact that all sensors achieved clinically acceptable performance suggests that the AI system is relatively insensitive to hardware variability. This is a significant strength, as variability in imaging hardware is a common challenge in deploying AI solutions across multiple dental clinics. Prior research has highlighted that AI models trained on heterogeneous datasets can maintain stable performance across imaging devices, provided that preprocessing and normalization techniques are appropriately implemented [12,17].

Another important aspect of this study is the comparison of AI performance for primary versus secondary caries. The system demonstrated similar sensitivity for both lesion types, although the positive predictive value was slightly lower for secondary caries. This finding is consistent with previous literature showing that secondary caries can be more challenging to detect radiographically due to restorative material artifacts, marginal gaps, or radiopaque restorations that obscure lesion boundaries [26]. Schwendicke et al. noted that AI systems may have reduced accuracy in detecting secondary lesions adjacent to restorations compared with primary lesions in intact enamel or dentin [9]. The slightly lower predictive value observed in the present study likely reflects these inherent diagnostic challenges rather than algorithmic deficiencies.

From a clinical perspective, the extremely high negative predictive values observed for both BW and IOPA radiographs are particularly noteworthy. A high NPV indicates that when the AI system predicts the absence of caries, clinicians can be highly confident in that assessment. This characteristic is valuable for screening applications, where minimizing missed lesions is critical. Previous studies evaluating AI in radiographic interpretation have emphasized that high specificity and NPV can help reduce unnecessary interventions while maintaining diagnostic safety [7]. The performance profile observed in this study, therefore, supports the potential role of AI as a decision-support tool that enhances diagnostic confidence and workflow efficiency.

The findings also align with broader trends in medical imaging, where deep learning-based diagnostic tools increasingly demonstrate performance comparable to expert clinicians. A landmark review by Alnaggar et al. highlighted that CNN-based systems often achieve human-level accuracy in image classification tasks across multiple medical specialties [17]. In dentistry, similar advancements have been reported for the detection of periodontal bone loss, periapical lesions, and tooth segmentation [27]. The present study contributes to this growing evidence by demonstrating strong AI performance, specifically for caries detection across multiple radiographic modalities and technical conditions.

Despite these strengths, several limitations should be considered. First, the study was retrospective, and although the dataset included diverse images, prospective clinical validation is necessary to confirm real-world performance. AI systems may perform differently when integrated into clinical workflows where image quality, patient positioning, and operator variability are less controlled. Second, the reference standard relied on expert consensus rather than histological confirmation. While this approach is common in radiographic research, it may introduce some degree of subjective interpretation bias [4]. Third, although the dataset included multiple sensor types, performance could vary further across other imaging systems not represented in the study. Future research should include multicenter prospective trials and external validation datasets to further confirm generalizability. Also, another limitation of our research work was that without multiple case comparisons and the multi-reader evaluation of radiologists which include MRMC (Multiple reader multiple cases) the validation of BDCA AI software is certainly a drawback. The other point was regarding the lack of caries stratification, in our AI software. Although it has been trained with binary classification, the categorization of caries lesions according to the severity or depth is still lacking. A key methodological limitation was the low surface level caries prevalence (3.73% in BW and 3.97% in IOPA). While this distribution mirrors typical outpatient screening demographics, it introduces spectrum bias. High proportions of TRUE NEGATIVE (TN) surfaces naturally inflate specificity (˂0.98) and NEGATIVE PREDICTIVE VALUE (NPV). Consequently, metrics must be interpreted with caution. Future prospective evaluations should incorporate high-risk clinical cohorts or enriched datasets to rigorously test the software’s POSITIVE PREDICTIVE VALUE (PPV) under high disease burden scenarios.

In addition, the integration of AI into dental practice raises considerations regarding clinician acceptance, workflow integration, and regulatory oversight. Studies have shown that AI decision-support tools can improve diagnostic accuracy when used alongside clinicians, but effectiveness depends on appropriate training, interface design, and understanding of system limitations [28,29]. Therefore, future investigations should examine not only standalone AI performance, but also clinician–AI collaborative performance in real clinical scenarios.

## 6. Conclusions

Overall, the results of this study demonstrate that the evaluated **Better Diagnostics Caries Assist (BDCA) Version 1.0** AI system provides highly accurate detection of dental caries on both bitewing and intraoral periapical radiographs. The consistent performance across demographic subgroups, sensor technologies, and lesion types indicates strong robustness and clinical applicability. When interpreted alongside existing literature, these findings reinforce the growing consensus that AI-assisted radiographic interpretation can enhance diagnostic reliability and efficiency in dental practice. Continued prospective validation and integration studies will be essential to fully realize the potential of AI-based caries detection in routine clinical care.

## Figures and Tables

**Table 1:** Dataset representing variables to assess the BDCA AI software for IOPA and bitewing.

S.N.O	Variable	Bitewings	IOPA
1.	Total no of participants	316 Females, 298 male patients	369 Females, 315 male patients
2.	Total no of radiographs	614	684
3.	Total no of tooth surfaces involved	15,687	9253
4.	Absolute no of True Positives (Carious tooth) True Negative (Non Carious tooth)	585 True positive 15,102 true negatives	367 True positive 8635 true negatives

**Table 2:** Overall diagnostic performance of BW and IOPA images.

Modality	Sensitivity (95% CI)	Specificity (95% CI)	PPV (95% CI)	NPV (95% CI)
BW	0.892 (0.8650–0.9147)	0.995 (0.9931–0.9956)	0.863 (0.8326–0.8883)	0.996 (0.9946–0.9968)
IOPA	0.882 (0.8503–0.9075)	0.991 (0.9886–0.9930)	0.876 (0.8452–0.9017)	0.992 (0.9890–0.9935)

Values are presented as point estimate (95% confidence interval). Adjusted confidence intervals were calculated using generalized estimating equations (GEE) to account for the clustering of multiple carious lesions per image. Abbreviations: BW = bitewing; IOPA = intraoral periapical; PPV = positive predictive value; NPV = negative predictive value; CI = confidence interval.

**Table 3:** Diagnostic performance by age group.

Modality	Age Group	Sensitivity (95% CI)	Specificity (95% CI)	PPV (95% CI)	NPV (95% CI)
BW	18–40	0.897 (0.8468–0.9325)	0.994 (0.9905–0.9960)	0.860 (0.8001–0.9042)	0.996 (0.9932–0.9972)
BW	41–60	0.881 (0.8306–0.9183)	0.995 (0.9926–0.9967)	0.865 (0.8105–0.9063)	0.996 (0.9936–0.9971)
BW	>60	0.901 (0.8497–0.9355)	0.994 (0.9921–0.9962)	0.862 (0.8115–0.9013)	0.996 (0.9939–0.9976)
IOPA	18–40	0.906 (0.8557–0.9394)	0.994 (0.9898–0.9959)	0.906 (0.8550–0.9397)	0.994 (0.9898–0.9959)
IOPA	41–60	0.885 (0.8302–0.9232)	0.989 (0.9842–0.9928)	0.868 (0.8138–0.9081)	0.991 (0.9858–0.9941)
IOPA	>60	0.854 (0.7849–0.9036)	0.991 (0.9861–0.9940)	0.859 (0.7945–0.9053)	0.991 (0.9853–0.9939)

**Table 4:** Diagnostic performance by sex.

Modality	Sex	Sensitivity (95% CI)	Specificity (95% CI)	PPV (95% CI)	NPV (95% CI)
BW	Male	0.875 (0.8341–0.9063)	0.994 (0.9916–0.9954)	0.851 (0.8098–0.8853)	0.995 (0.9930–0.9963)
BW	Female	0.910 (0.8706–0.9388)	0.995 (0.9931–0.9966)	0.874 (0.8274–0.9096)	0.997 (0.9950–0.9978)
IOPA	Male	0.901 (0.8526–0.9348)	0.992 (0.9875–0.9943)	0.882 (0.8316–0.9193)	0.993 (0.9893–0.9955)
IOPA	Female	0.866 (0.8209–0.9006)	0.991 (0.9872–0.9932)	0.871 (0.8295–0.9034)	0.990 (0.9865–0.9930)

**Table 5:** Diagnostic performance by sensor type for BW and IOPA images.

Sensor	BW Sens (95% CI)	IOPA Sens (95% CI)	BW Spec (95% CI)	IOPA Spec (95% CI)	BW PPV (95% CI)	IOPA PPV (95% CI)	BW NPV (95% CI)	IOPA NPV (95% CI)
Apex	0.933 (0.8720–0.9656)	0.925 (0.8405–0.9663)	0.994 (0.9889–0.9964)	0.993 (0.9856–0.9968)	0.838 (0.7548–0.8973)	0.915 (0.8280–0.9600)	0.998 (0.9948–0.9989)	0.994 (0.9865–0.9974)
Carestream	0.900 (0.8349–0.9412)	0.859 (0.7361–0.9305)	0.995 (0.9901–0.9973)	0.992 (0.9812–0.9964)	0.910 (0.8418–0.9506)	0.887 (0.7804–0.9456)	0.994 (0.9892–0.9968)	0.989 (0.9776–0.9950)
Dentimax	0.833 (0.7237–0.9052)	0.891 (0.8094–0.9406)	0.998 (0.9957–0.9991)	0.994 (0.9892–0.9967)	0.915 (0.8249–0.9614)	0.891 (0.8105–0.9402)	0.996 (0.9924–0.9977)	0.994 (0.9892–0.9967)
Dexix	0.924 (0.8258–0.9690)	0.828 (0.7371–0.8919)	0.994 (0.9890–0.9967)	0.984 (0.9746–0.9901)	0.830 (0.7169–0.9034)	0.821 (0.7301–0.8862)	0.998 (0.9940–0.9990)	0.985 (0.9749–0.9909)
Vatech	0.930 (0.8596–0.9665)	0.871 (0.7566–0.9366)	0.993 (0.9860–0.9965)	0.988 (0.9770–0.9939)	0.894 (0.8176–0.9410)	0.847 (0.7413–0.9147)	0.996 (0.9899–0.9980)	0.990 (0.9792–0.9955)
Jazz	0.841 (0.7248–0.9143)	0.933 (0.8706–0.9661)	0.994 (0.9899–0.9965)	0.993 (0.9868–0.9963)	0.815 (0.6975–0.8943)	0.915 (0.8506–0.9533)	0.995 (0.9911–0.9972)	0.995 (0.9888–0.9973)
SHICK	0.860 (0.7797–0.9149)	0.863 (0.7309–0.9360)	0.992 (0.9871–0.9951)	0.992 (0.9838–0.9965)	0.831 (0.7525–0.8889)	0.863 (0.7309–0.9360)	—	0.992 (0.9838–0.9965)

**Table 6:** Diagnostic performance by lesion type (IOPA and bitewing images).

Lesion Type	Sensitivity (95% CI)	PPV (95% CI)
Primary decay	0.883 (0.8441–0.9133)	0.837 (0.7989–0.8690)
Secondary decay	0.879 (0.8221–0.9197)	0.758 (0.6934–0.8132)

## Data Availability

The data will be made available on request from corresponding author.
